# Robust identification key predictors of short- and long-term weight status in children and adolescents by machine learning

**DOI:** 10.3389/fpubh.2024.1414046

**Published:** 2024-09-24

**Authors:** Hengyan Liu, Yang Leng, Yik-Chung Wu, Pui Hing Chau, Thomas Wai Hung Chung, Daniel Yee Tak Fong

**Affiliations:** ^1^School of Nursing, The University of Hong Kong, Pokfulam, Hong Kong SAR, China; ^2^Department of Electrical and Electronic Engineering, The University of Hong Kong, Pokfulam, Hong Kong SAR, China; ^3^Family and Student Health Branch, Department of Health, Kwun Tong, Kowloon, Hong Kong SAR, China

**Keywords:** child, obesity, machine learning, feature selection, feature stability

## Abstract

**Background:**

Early identification of high-risk individuals for weight problems in children and adolescents is crucial for implementing timely preventive measures. While machine learning (ML) techniques have shown promise in addressing this complex challenge with high-dimensional data, feature selection is vital for identifying the key predictors that can facilitate effective and targeted interventions. This study aims to utilize feature selection process to identify a robust and minimal set of predictors that can aid in the early prediction of short- and long-term weight problems in children and adolescents.

**Methods:**

We utilized demographic, physical, and psychological wellbeing predictors to model weight status (normal, underweight, overweight, and obese) for 1-, 3-, and 5-year periods. To select the most influential features, we employed four feature selection methods: (1) Chi-Square test; (2) Information Gain; (3) Random Forest; (4) eXtreme Gradient Boosting (XGBoost) with six ML approaches. The stability of the feature selection methods was assessed by Jaccard's index, Spearman's rank correlation and Pearson's correlation. Model evaluation was performed by various accuracy metrics.

**Results:**

With 3,862,820 million student-visits were included in this population-based study, the mean age of 11.6 (SD = 3.64) for the training set and 10.8 years (SD = 3.50) for the temporal test set. From the initial set of 38 predictors, we identified 6, 9, and 13 features for 1-, 3-, and 5-year predictions, respectively, by the best performed feature selection method of Chi-Square test in XGBoost models. These feature sets demonstrated excellent stability and achieved prediction accuracies of 0.82, 0.73, and 0.70; macro-AUCs of 0.94, 0.86, and 0.83; micro-AUCs of 0.96, 0.93, and 0.92 for different prediction windows, respectively. Weight, height, sex, total score of self-esteem, and age were consistently the most influential predictors across all prediction windows. Additionally, several psychological and social wellbeing predictors showed relatively high importance in long-term weight status prediction.

**Conclusions:**

We demonstrate the potential of ML in identifying key predictors of weight status in children and adolescents. While traditional anthropometric measures remain important, psychological and social wellbeing factors also emerge as crucial predictors, potentially informing targeted interventions to address childhood and adolescence weight problems.

## 1 Introduction

The prevalence of weight problems, particularly among younger children, has been steadily increasing, raising significant concerns for public health (1). Overweight and obesity in children are strongly associated with major non-communicable diseases and can have detrimental effects on physical health (2). Additionally, children who are overweight or obese may face social challenges, including difficulties in making friends and being teasing by peers (3). Furthermore, these weight problems tend to persist into adulthood (4), thereby increasing the risk of long-term health complications. The prevalence of overweight and obesity has been on the rise in Hong Kong (5). Survey reported that about one in five primary students were overweight or obese for 2018/2019 school year, with a further surge to 24.1% reported during the COVID-19 pandemic in 2020 (6, 7). Being underweight is another weight problem that cannot be ignored, as it has been linked to psychiatric disorders, osteoporosis, scoliosis, and delayed pubertal development (8). In Hong Kong, the prevalence of underweight and severely underweight among primary 1 (P1, equivalent to United States Grade 1) students, was found to be 11.3 and 5.4% in boys, and 16.4 and 5.9% in girls, respectively, in 2006–2007 (9). The proportion of underweight children among all primary school students was reported to be 6.7% for boys and 11.9% for girls in 2014 (10). Given the urgent need to address weight problems in childhood and adolescence, early intervention is crucial (11). Consequently, accurate predictive models that can classify a child's future weight status would be valuable tools for early intervention.

The rapid advancements in data storage and capture capabilities have led to abundance of data in healthcare (12). Feature selection is a common approach to address the challenge of excessive data and unnecessary features (13, 14). By selecting the most relevant features, effective feature selection can reduce computing time (15), improve the overall performance of machine learning (ML) algorithms (16), and potentially insights into underlying biological processes (17). Previous studies among pediatric population have explored feature reduction in binary prediction of obesity and (or) overweight, primarily using features related to birth, infant, or parental measurements for early childhood or short-term predictions (18–23). One study has utilized laboratory measurements for long-term prediction (24). However, laboratory tests are less viable and cost-effective for the general population, limiting their clinical applicability. In addition, previous studies often reported the most important variables without thoroughly discussing predictive improvement and clinical application. Moreover, the use of single feature method in previous studies may overlook the importance of comparing different feature selection methods and evaluating the stability of the identified features. High stability is equally important as high prediction accuracy (25, 26).

To address the current gaps, we aimed to identify robust predictors for short- and long-term weight status in children and adolescents. We would use multiclass ML models to analyze various demographic, physical and psychological wellbeing features to determine which predictors contribute most significantly. By identifying the robust predictors, we can provide clinicians and health providers with a simplified and effective approach to predict future weight status in pediatric populations. This may ultimately improve clinical decision-making and inform interventions to prevent and treat childhood weight problems.

## 2 Materials and methods

### 2.1 Study design and setting

This was a population-based retrospective cohort study of students enrolled from P1 to secondary 6 (S6, equivalent to United States Grade 12) in Hong Kong during the academic cohorts of 1995/1996 to 2019/2020. Data were sourced from the Student Health Service (SHS) of the Department of Health, which has been providing annual voluntary health assessments to primary and secondary students across Hong Kong since the academic year of 1995/1996. To be included, students had to have participated in the SHS measurement for at least 2 years. We included all grades from 1995/1996 to 2014/2015 to allow for at least 1 year of follow-up, as the student lifestyle assessment questionnaire was updated in 2015/2016. More details about SHS health assessment program can be found elsewhere (27).

### 2.2 Data collection

We collected 38 predictors from the SHS data, including demographic information (age, sex), socioeconomic status indicators (housing type, home and school district, parental occupation and education levels), physical examination data (weight, height), and biennial records of physical and psychosocial measurements (details in Supplementary Table 1) (28–34).

### 2.3 Prediction outcome

Weight status was classified as underweight, normal weight, overweight, or obese based on the age- and sex-specific body mass index (BMI, expressed in kg/m^2^) reference standards developed by the International Obesity Task Force (IOTF). The IOTF provided a comprehensive set of BMI cut-off values for classifying weight status in children and adolescents aged 2–18 years, with cut-offs specified for each sex at 6-month age intervals (35, 36).

### 2.4 Data preparation

To prepare the data for analysis, we first removed responses from students with a lie self-esteem score ≤ 2, which was designed to assess reliability. We then ordered the categorical socioeconomic status variables based on Hong Kong median monthly domestic household income statistics and used one-hot encoding for the sex variable.

To ensure robustness of our analysis, the data sets for training and temporal test phases were partitioned according to students' enrollment academic years. Specifically, we used the academic cohorts of 1995/96 to 2009/10 for the training phase, and the academic cohorts of 2010/11 to 2014/15 for the temporal test phase. For each of these training and temporal test data sets, we examined the 1-, 3-, and 5-year prediction on children with the corresponding measurements available.

The missing data in our study was primarily due to the biennial nature of the physical and psychological measurements collected through the SHS questionnaires. To ensure complete data for ML analysis, we applied the k-Nearest Neighbor (k-NN) imputation method to fill the gaps in the missing years. The k-NN imputation approach uses the information from the nearest data points to estimate the missing values, effectively handling both continuous and categorical variables (37). We initially selected only the visit records without missing questionnaire data to perform the feature selection process. We then compared the results with those from data imputed using the *k*-NN method with different *k*-values (*k* =3 and 5). The feature rankings remained largely unchanged. Thus, we have taken *k* = 3 with lower computing intensity. The detailed results are presented in Supplementary Table 2. The imputation was implemented using the “KNNImputer” module from the scikit-learn library in Python.

All the data were standardized to a 0–1 scale to facilitate comparisons and eliminate scale-related biases.

### 2.5 Feature selection

#### 2.5.1 Feature selection methods

To identify the most relevant features for predicting short- and long-term weight status, we attempted several feature selection methods. Feature selection methods can be broadly categorized into: Filter, Wrapper, and Embedded. Filter methods evaluate the relevance of features based on their statistical properties. They can be filter-univariate and filter-multivariate, with the latter being more computationally intensive and not as scalable as filter-univariate (38). Wrapper methods evaluate different feature subsets and select the one that performs best. Thus, they are also computationally expensive and time-consuming (14). Embedded methods integrate the feature selection process during the model development, allowing the model to select the most relevant features (39). Therefore, to allow efficient feature selection, we opted for filter-univariate and embedded methods.

Among the filter-univariate methods, we adopted the Chi-Square test and Information Gain (40, 41). The Chi-Square test evaluates the relationship between a feature and the target variable while Information Gain measures the reduction in entropy (or uncertainty) about the target variable achieved by knowing the value of a feature (39, 40). These approaches can effectively capture both linear and non-linear relationships between features and the target variable and are less sensitive to outliers (41). For the embedded feature selection techniques, we employed the Random Forest (RF) and eXtreme Gradient Boosting (XGBoost) (38). These are tree-based algorithms that provide built-in measures of feature importance, which are calculated based on the decrease in impurity (Gini importance) for RF (42), and the gain in predictive performance for XGBoost (43), when a feature is used for tree splitting. The inclusion of these embedded methods allowed us to leverage the model-specific knowledge to further refine the feature subset.

#### 2.5.2 Feature selection analysis

Due to the high computational intensity of model using the entire sets, we randomly selected 10% of the subjects from the training subsets 10 times, without replacement, creating 10 sub-training samples for each of the 1-, 3-, and 5-year prediction periods.

For each sub-training sample, we applied the four feature selection methods to obtain four lists of features, from 1 to 38. To assess the stability of each feature list, we calculated three stability indices: (1) Jaccard's index, which measured stability by subset and was calculated by the amount of overlap between the overall subset of selected features; (2) Spearman's rank correlation coefficient, which measured stability by features' importance rank; and (3) Pearson's correlation coefficient, which measured stability by features' importance value (provided as “weight”) (14). We then obtained the average stability indices across the 10 sub-training samples for each fixed number of features.

The Synthetic Minority Oversampling Technique (SMOTE) was employed to handle imbalanced data structure to the training set within each cross-validation fold before model development (44). SMOTE generates synthetic examples of the minority classes by interpolating between existing minority class samples. This helps to balance the class distributions and improve the model's ability to learn from the minority classes (45). SMOTE was implemented using the default parameters in the “imbalanced-learn” and scikit-learn libraries in Python.

We attempted several multiclass ML prediction approaches for each of the random sub-training sample to predict weight status at 1, 3, and 5 years. The ML models included decision tree (DT) (46), RF (43), Support Vector Machine (SVM) (47), k-NN (48), XGBoost (49), and logistic regression (LR). To optimize the performance of these models, we conducted a grid search-based hyperparameter tuning procedure within a 10-fold cross-validation framework. The averaged correct classification rate, accuracy, macro- Area Under the Receiver Operating Characteristic Curve (macro-AUC), and micro-AUC were used to compare the performance of the tuned models for each prediction period. Macro-AUC, which calculates the AUC for each class individual and gives the equal importance. Micro-AUC combines the true positives, false positives, true negatives, and false negatives across all classes to calculate a single AUC value, which more influenced by the performance on the majority class.

During the temporal test phase, we evaluated the predictive performance of the top-performing ML models using the 10 feature sets identified during the preceding training phase, for each fixed number of features. The best performing hyperparameter values were determined through 10-fold cross-validation. The specific hyperparameters tuned for each ML model are detailed in Supplementary Table 3. We calculated the overall performance metrics by averaging the accuracy, macro-AUC, and micro-AUC across the folds. Additionally, we averaged the precision, recall, and F1-score for the abnormal weight status predictions, as these metrics provide complementary insights into the model's performance. The precision and recall are conceptually equivalent to the sensitivity and positive predictive value, and the F1 score is the harmonic mean of precision and recall. The optimal number of features for each ML approach were determined by a sequential process: (1) Identify the minimal number of features such that adding more features would not substantially improve the averaged value of accuracy, micro-AUC, and macro-AUC (defined as a change < 0.05); and (2) Increase the number of features, if needed, until additional features would not substantially increase the three stability indices.

All above feature selection methods and ML models were performed using the scikit-learn and “xgboost” libraries in Python software (version 3.10).

## 3 Results

### 3.1 Participants characteristics

**Figure 1:**
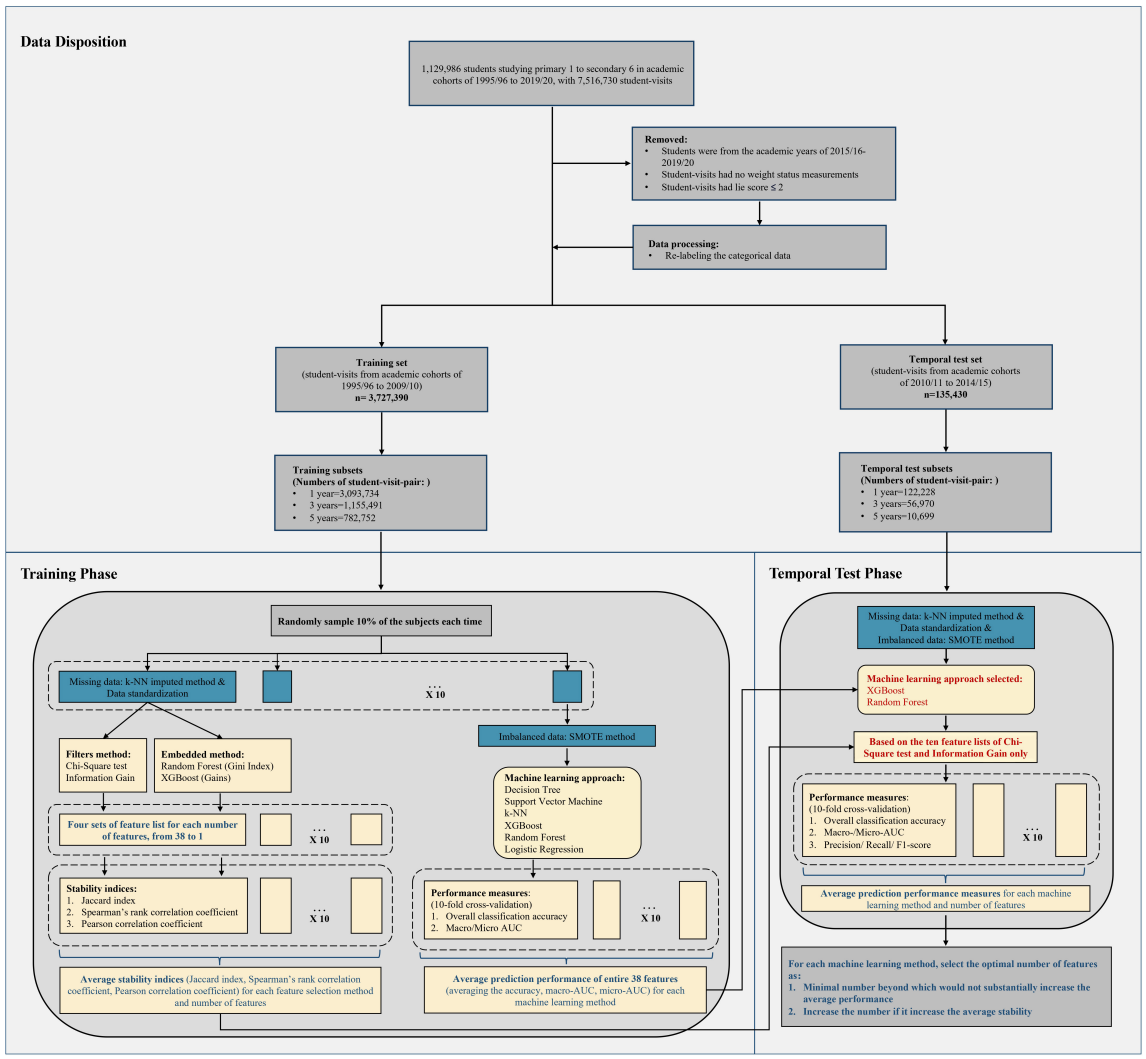
Flow chart of the study design.

During the study period, 3,862,820 student-visits were included for weight status prediction and feature selection. Their disposition and details of the study design are displayed in Figure 1. Of these, 3,727,390 student-visits were assigned to the training set, with a mean age of 11.6 years (Standard Deviation, SD = 3.64), and 135,430 student-visits were assigned to the temporal test set, with a mean age of 10.8 years (SD = 3.50). In the training set, 1,953,152 (52.4%) were male, while in the temporal test set, 65,684 (48.5%) were male. The prevalence of underweight, overweight, and obese in the training set was 18.5, 11.7, and 2.9%, respectively. In the temporal test set, the prevalence was 13.7, 11.7, and 2.0%, respectively. We identified 3,093,734 (83.0%), 1,155,491 (31.0%), and 782,752 (21.0%) student visit-pairs in the training set for 1-, 3-, and 5-year predictions, and 122,228 (90.3%), 56,970 (42.1%), and 10,699 (7.9%) student visit-pairs, respectively, in temporal test set. Table 1 presents the characteristics of all cohorts and displays the baseline characteristics of the training and temporal test sets. Supplementary Table 4 shows the baseline socioeconomic characteristics of training and temporal test sets.

**Table 1:** Characteristics of baseline training and temporal test sets.

**Characteristics**	**Training set**	**Temporal test set (academic cohorts of 2010/11–2014/15)**
	**N_1_ = 3,727,390**	**N_2_ = 135,430**
**Numbers of students followed at**		
1 year	3,093,734 (83.0)	122,228 (90.3)
3 years	1,155,491 (31.0)	56,970 (42.1)
5 years	782,752 (21.0)	10,699 (7.9)
**Sex**		
Male	1,953,152 (52.4)	65,684 (48.5)
Female	1,774,238 (47.6)	69,746 (51.5)
**Age**, *mean (SD), years*	11.6 (3.64)	10.8 (3.50)
**Weight status**		
Normal	2,493,624 (66.9)	98,322 (72.6)
Underweight	689,567 (18.5)	18,554 (13.7)
Overweight	436,105 (11.7)	15,845 (11.7)
Obese	108,094 (2.9)	2,709 (2.0)
**Weight**, *mean (SD), kg*	37.3 (12.71)	38.3 (14.10)
**Height**, *mean (SD), cm*	141.5 (15.33)	144.5 (17.34)
**Breakfast eating habit**		
Missing value	2,221,524 (59.6)	82,341 (60.8)
Home	1,129,399 (30.3)	45,369 (33.5)
Rarely at home	294,464 (7.9)	4,605 (3.4)
No breakfast	82,003 (2.2)	3,115 (2.3)
**Sweetness preference during past 7 days**		
Missing value	2,273,708 (61.0)	101,301 (74.8)
0–3 times	674,658 (18.1)	19,637 (14.5)
4–6 times	361,557 (9.7)	10,293 (7.6)
Once daily	167,733 (4.5)	3,386 (2.5)
2 times or above daily	249,735 (6.7)	813 (0.6)
**Junk food intake habits**		
Missing value	2,281,162 (61.2)	101,288 (74.8)
Every day	290,736 (7.8)	3,414 (2.5)
Occasionally	603,837 (16.2)	18,095 (13.4)
Rarely	369,012 (9.9)	11,836 (8.7)
Never	182,642 (4.9)	797 (0.6)
**Fruit/vegetable intake**		
Missing value	2,310,982 (62.0)	101,402 (74.9)
At least thrice a day	130,459 (3.5)	6,601 (4.9)
Once or twice a day	752,933 (20.2)	23,330 (17.22)
Once every few days	506,925 (13.6)	3,300 (2.4)
Less than once a week	26,092 (0.7)	797 (0.6)
**Milk consumption habit**		
Missing value	2,273,708 (61.0)	101,288 (74.8)
At least once a day	383,921 (10.3)	8,536 (6.3)
Once every few days	495,743 (13.3)	13,998 (10.3)
Less than once a week	275,827 (7.4)	5,918 (4.4)
Never	298,191 (8.0)	5,690 (4.2)
**Frequency of aerobic exercise (for primary school)**		
Missing value	2,396,712 (64.3)	101,629 (75.0)
At least thrice a week	249,735 (6.7)	7,170 (5.3)
Once or twice a week	786,479 (21.1)	16,730 (12.4)
Less than once a week	190,097 (5.1)	7,739 (5.7)
Never	104,367 (2.8)	2,162 (1.6)
**Frequency of aerobic exercise (for secondary school)**		
Missing value	2,840,271 (76.2)	115,855 (85.5)
Never or rarely	167,733 (4.5)	2,845 (2.1)
Once or thrice a month	454,742 (12.2)	4,552 (3.4)
Once a week	167,733 (4.5)	6,373 (4.7)
At least twice a week	96,912 (2.6)	5,804 (4.3)
**Hours of aerobic exercise**		
Missing value	2,102,247 (56.4)	82,282 (60.8)
More than an hour	603,837 (16.2)	18,323 (13.5)
Half to one hour	652,293 (17.5)	18,095 (13.4)
Less than half an hour	268,372 (7.2)	12,177 (9.0)
Zero	100,640 (2.7)	4,552 (3.4)
**Daily hours of TV viewing**		
Missing value	2,083,611 (55.9)	101,288 (74.8)
Less than an hour	294,464 (7.9)	4,666 (3.4)
One to 2 h	756,660 (20.3)	11,722 (8.7)
Two to 4 h	309,373 (8.3)	14,567 (10.8)
More than 4 h	283,282 (7.6)	3,187 (2.4)
**SEI score**^a^, *mean (SD)*		
Total	37.0 (14.35)	39.8 (15.13)
General	14.9 (5.96)	15.5 (6.37)
Social	6.5 (2.82)	6.7 (3.00)
School-related	7.1 (2.84)	7.5 (3.26)
Lie (exclude score ≤ 2)	5.9 (2.71)	6.1 (2.84)
Parent-related	8.6 (2.87)	9.0 (3.74)
**YSR score**^b^, *mean (SD)*		
Total	32.4 (14.92)	31.5 (14.60)
Withdrawal	3.8 (1.91)	3.7 (1.89)
Somatic complaints	3.0 (1.56)	2.8 (1.50)
Anxious/depressed	5.5 (3.2)	5.3 (3.00)
Social problems	4.5 (1.96)	4.4 (1.91)
Thought problems	2.6 (1.44)	2.2 (1.10)
Attention problems	4.7 (2.53)	4.8 (2.52)
Delinquent behavior	3.3 (1.43)	3.0 (1.38)
Aggressive behavior	3.6 (1.42)	3.1 (1.35)
**RBQ score**^c^, *mean (SD)*		
Total	9.4 (3.15)	9.4 (3.16)
Conduct	1.5 (0.75)	1.1 (0.72)
Emotion	1.4 (0.79)	1.4 (0.83)
Hyperactivity	1.8 (0.91)	1.4 (0.81)

Data are n (%) unless otherwise stated.

^a^SEI, Culture Free Self-Esteem Inventory for Children Questionnaire.

^b^YSR, Youth Self-Report.

^c^RBQ, Rutter Behavior Questionnaire.

### 3.2 Features selected for prediction

**Figure 2:**
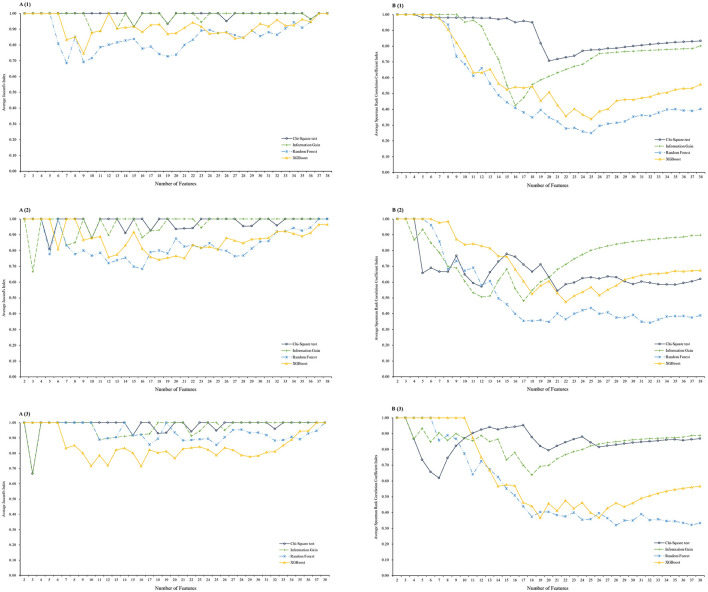
Average stability indices for different prediction windows. **(A)** Average Jaccard's Index for stability by subset, **(B)** Average Spearman's rank correlation coefficient Index for stability by rank; (1) on 1 year prediction window, (2) on 3-year prediction window, and (3) on 5-year prediction window; *XGBoost*, eXtreme Gradient Boosting feature selection method.

Figure 2 presents the averaged stability indices of the features selected using the Chi-Square test, Information Gain, RF, and XGBoost feature selection methods. The Pearson's correlation coefficient, which measured the stability of weight, consistently showed high values approaching 1 across all methods, and was not presented in the plots. All feature selection methods exhibited a nearly “*U*-shape” stability of rank as the feature size increased, indicating that the top and tail features were more robust. The Chi-Square test had less stable training results for the 3-year prediction. Overall, the Chi-Square test and Information Gain produced more stable feature subsets and ranks for all 1-, 3-, and 5-year weight status prediction. The features lists obtained from the Chi-Square Test and Information Gain were more stable than those from the RF and XGBoost, and thus only considered them in the temporal test phase.

**Figure 3:**
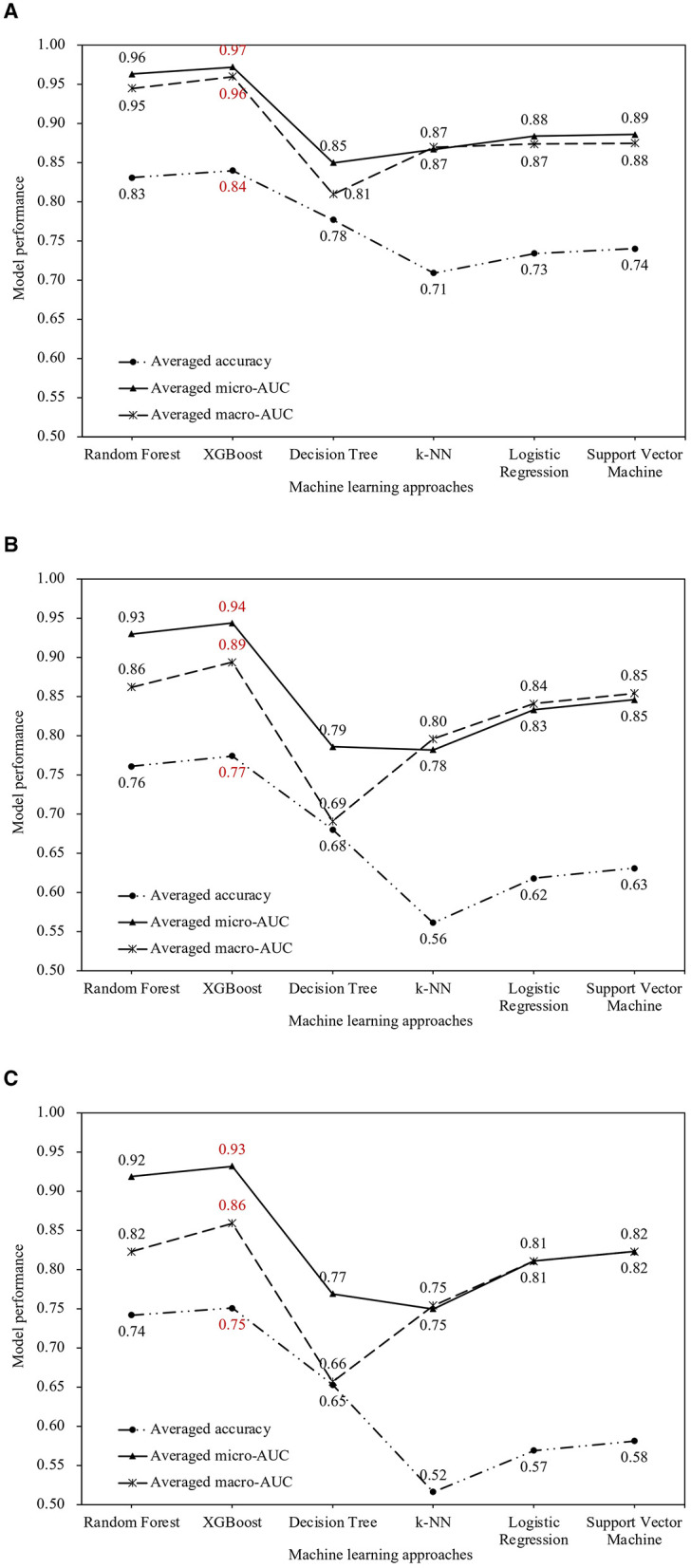
Prediction performance of different machine learning methods using 38 features. *XGBoost*, eXtreme Gradient Boosting, *k-NN*, k-Nearest Neighbors; *AUC*, Area Under the Receiver Operating Characteristic Curve. **(A)** 1-year prediction. **(B)** 3-year prediction. **(C)** 5-year prediction.

Figure 3 shows the averaged accuracy, macro- and micro-AUC of each prediction ML model and the conventional statistic method by LR. For the 1-year prediction, XGBoost achieved an overall accuracy of 0.84, a macro-AUC of 0.96, and a micro-AUC of 0.97. The 3-year prediction results showed XGBoost's accuracy at 0.77, macro-AUC at 0.89, and micro-AUC at 0.94. The 5-year prediction performance of XGBoost was consistently excellent, with an accuracy of 0.75, macro-AUC of 0.86, and micro-AUC of 0.93. RF attained accurate prediction, with 1-year accuracies of 0.83, macro-AUC of 0.95, and micro-AUC of 0.96. For the 3- and 5-year predictions, RF achieved accuracies of 0.76 and 0.74, macro-AUCs of 0.86 and 0.82, and micro-AUCs of 0.93 and 0.92, respectively. In contrast, the performance of LR was relatively lower, with 1-year accuracies of 0.73, macro-AUC of 0.87, and micro-AUC of 0.88. The 3- and 5-year predictions by LR had accuracies of 0.62 and 0.57, macro-AUCs of 0.84 and 0.81, and micro-AUCs of 0.83 and 0.81, respectively.

**Figure 4:**
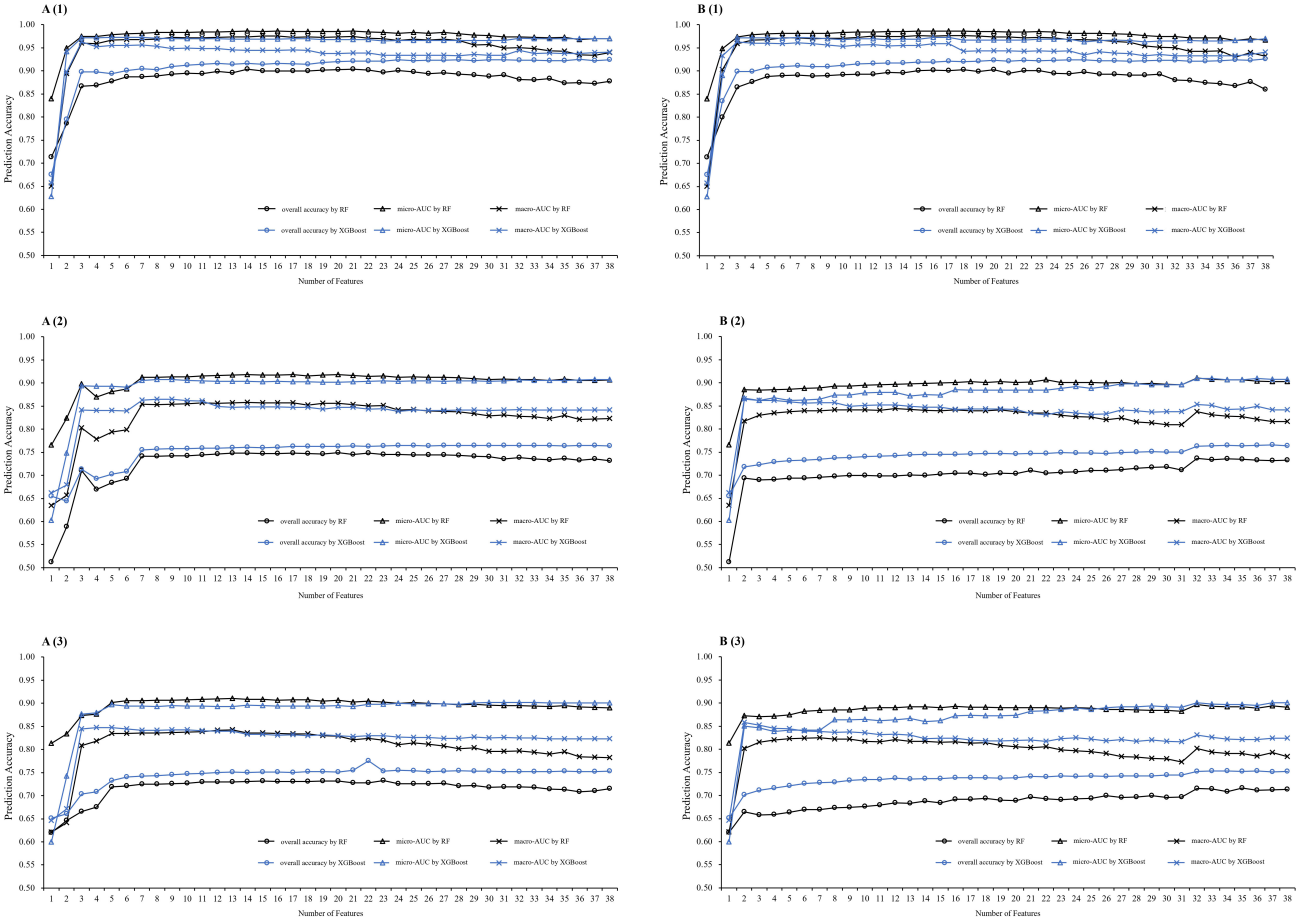
Model prediction performance at varying number of features. Model performance comparisons were conducted in the temporal test phase on different prediction windows. **(A)** By Chi-Square test feature selection method, **(B)** by Information Gain feature selection method; (1) on 1 year prediction window, (2) on 3-year prediction window, (3) on 5-year prediction window; *RF*, Random Forest model, *XGBoost*, eXtreme Gradient Boosting model; *AUC*, Area Under the Receiver Operating Characteristic Curve.

Figure 4 displays the XGBoost and RF models' performance at varying numbers of features. Feature lists obtained by Chi-Square test help the models consistently outperformed for all prediction windows. Based on the feature number determination strategies, we selected several minimized feature subsets for further comparison. For 1-year weight status prediction, we selected 6 features with good model performance and stability indices. For 3-year weight status prediction, we initially selected 7 features and then extended it to 9. For 5-year prediction, we initially selected 6 features and then extended it to 13. The Jaccard's index was nearly equal to 1 for the selected subsets, so Spearman's Rank Correlation Coefficient and test accuracy were considered.

**Figure 5:**
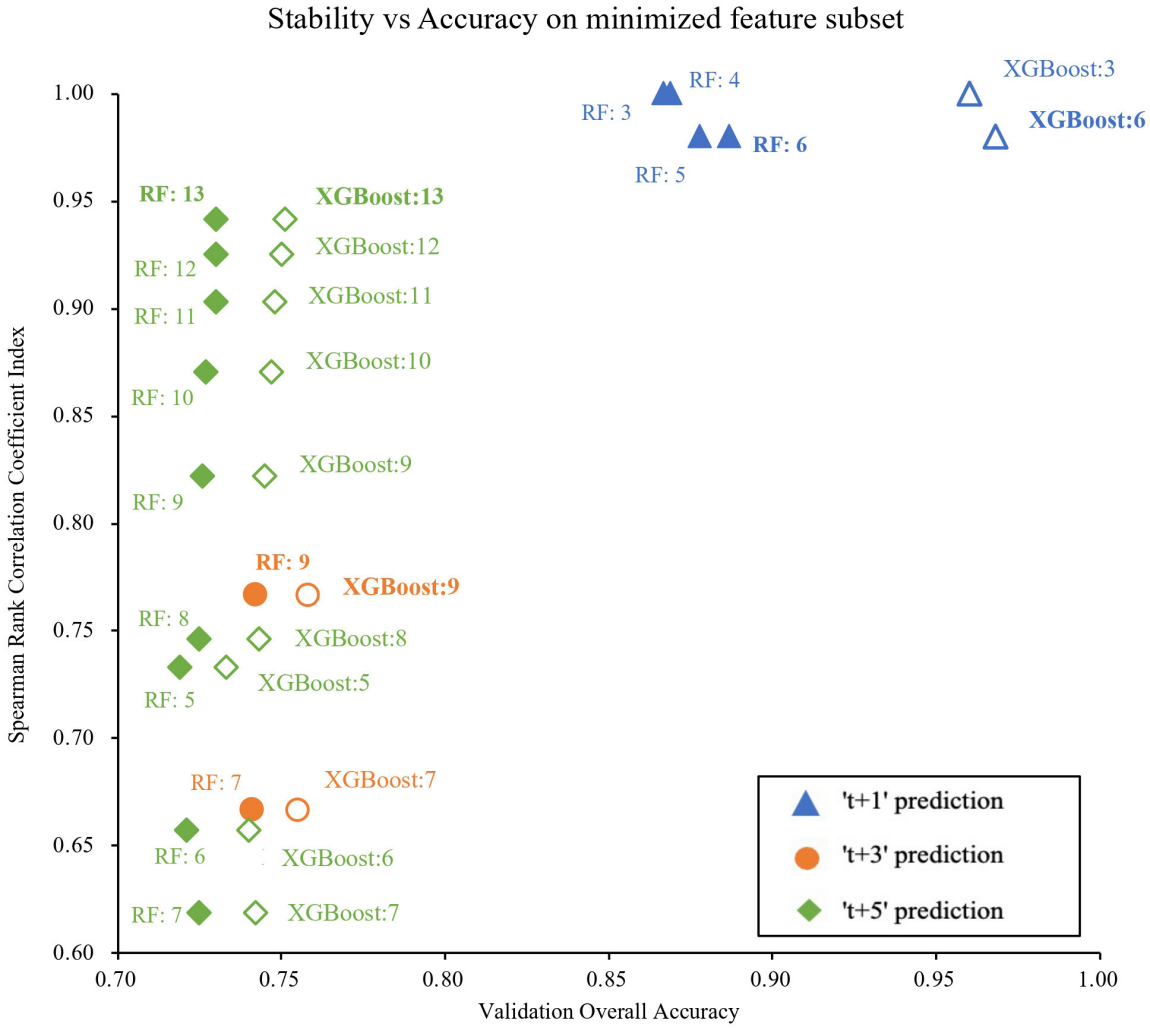
Plot of stability against overall accuracy when the Chi-Square test is used for feature selection. In the plot, each point shows with its applied model and the numbers of feature. *RF*, Random Forest model, *XGBoost*, eXtreme Gradient Boosting model.

Figure 5 shows the feature selection stability and their ML accuracy of the selected data subsets. We finally selected 6, 9, and 13 features for 1-, 3-, and 5-year weight status prediction, respectively. These final selected features enabled the classifiers of XGBoost and RF to achieve their best performance, and detailed prediction performance is presented in Table 2. Overall, XGBoost provided the higher accurate prediction with the same number of features for short- and long-term weight status and yielded the best estimates for the four weight status categories across different prediction windows. The accuracies of XGBoost were 0.82, 0.73, and 0.70; the macro-AUCs were 0.94, 0.86, and 0.83; and the macro-AUCs were 0.96, 0.93, and 0.82 for 1-, 3-, and 5-year predictions, respectively. Table 3 summarizes the most frequent features selected across all prediction windows. The final selected feature lists were consistent in each of the ten repeated training sessions. Interestingly, the 13 features selected for 5-year prediction included almost all the same features as for 1- and 3-year weight status prediction. Weight, sex, height, total score of SEI, and age were the commonly key predictors.

**Table 2:** Performance evaluations for machine learning models based on 10-fold cross-validation.

		**1 year prediction with 6 features**			**3-year prediction with 9 features**			**5-year prediction with 13 features**		
**Overall performance**	**Model**	**Accuracy**	**Macro-AUC**	**Micro-AUC**	**Accuracy**	**Macro-AUC**	**Micro-AUC**	**Accuracy**	**Macro-AUC**	**Micro-AUC**
	XGBoost	0.82	0.94	0.96	0.73	0.86	0.93	0.70	0.83	0.92
	RF	0.80	0.92	0.95	0.71	0.84	0.92	0.66	0.80	0.90
**Weight status**	**Model**	**Precision**	**Recall**	**F1-score**	**Precision**	**Recall**	**F1-score**	**Precision**	**Recall**	**F1-score**
Normal	XGBoost	0.87	0.87	0.87	0.79	0.85	0.82	0.76	0.81	0.79
	RF	0.85	0.85	0.85	0.77	0.83	0.80	0.73	0.80	0.77
Underweight	XGBoost	0.73	0.72	0.73	0.72	0.53	0.63	0.67	0.51	0.59
	RF	0.70	0.74	0.72	0.70	0.51	0.61	0.66	0.48	0.57
Overweight	XGBoost	0.73	0.74	0.73	0.59	0.60	0.59	0.52	0.53	0.52
	RF	0.71	0.70	0.70	0.55	0.55	0.55	0.47	0.48	0.47
Obese	XGBoost	0.76	0.75	0.76	0.52	0.61	0.56	0.49	0.57	0.53
	RF	0.73	0.66	0.70	0.49	0.55	0.52	0.37	0.40	0.38

XGBoost, eXtreme Gradient Boosting; RF, random forest; AUC, Area Under the Receiver Operating Characteristic Curve.

**Table 3:** Most frequently selected features ranking for each prediction window among the 10 times samples.

**Prediction window**	**1 year prediction**	**3-year prediction**	**5-year prediction**
Feature list	Weight	Weight	Weight
	Sex	Sex	Sex
	Height	Age	Height
	Age	Total score of YSR	Total score of YSR
	Total score of SEI	Total score of SEI	Age
	Thought score of YSR	Student's home district	Student's school district
		Height	Student's home district
		Student's school district	Total score of SEI
		General score of SEI	General score of SEI
			Aggressive score of YSR
			Mother occupation
			Total score of RBQ
			Delinquent score of YSR

SEI, Culture Free Self-Esteem Inventory for Children Questionnaire.

YSR, Youth Self-Report.

RBQ, Rutter Behavior Questionnaire.

## 4 Discussion

Our study, which utilized a large population-based cohort of children and adolescents, is unique in its thorough feature selection process to identify robust sets of predictors for short- and long-term weight status prediction. The selected easily accessible features not only offer valuable insights into the influential factors of weight problems in children and adolescents but also provide a useful tool for self-screening assessment. By identifying a small set of readily available predictors, healthcare providers, schools, and families can implement effective and targeted interventions to promote healthier behaviors and mitigate the development of long-term weight problems.

### 4.1 Key predictors identification

We selected 6, 9, and 13 out of 38 features for 1-, 3-, and 5-year weight status prediction, respectively, indicating a substantial decrease in the computational burden for further analysis. The top five features: weight, height, sex, total score of SEI, and age, were consistently ranked highly across repeated feature selections and different prediction time intervals. This consistency suggests that these variables are crucial predictors of weight problems. However, the accuracy of multiclass models decreased significantly when only the top four predictors were used, indicating the need for additional predictors from different aspects to enhance early weight problem prediction.

The results highlight the importance of psychological and emotion behavior factors in weight status prediction. The total score of SEI, reflecting a child's self-esteem, consistently demonstrated a high influence on weight status prediction. Previous epidemiological research has established an association between low self-esteem and weight problems (50–52). Moreover, longitudinal studies have also found that low baseline self-esteem and decreases in global self-esteem levels significantly predicted increased BMI over time in school children (52). Low self-esteem may be a significant predisposing factor, as individuals with low self-esteem may be more prone to engage in unhealthy behaviors such as overeating and avoiding physical activities (50). These findings underscore the need for early intervention in children and adolescents with low self-esteem to prevent or manage weight problems.

Our study identified several unconventional features contributed to short-term prediction accuracy. One of these, the “thought score of YSR,” which reflects thought problems such as disorganized ideas, delusions, and hallucinations, has been found to be associated with increased sleep problems, which, in turn, can lead to uncontrolled weight gain (53). Indeed, clinical evidence has shown that insufficient sleep or sleep difficulties are linked to higher levels of the hormone ghrelin, which increases appetite, and lower levels of hormone leptin, leading to decreased feelings of fullness, resulting in weight gain (54). In addition, the general score of SEI, total score of RBQ, and total score of YSR, especially “aggressive behavior” and “delinquent behavior” of YSR showed increased accuracy in predicting weight status over a 5-year period. Adolescents with emotional and behavioral problems are more susceptible to losing behavioral control, disordered eating, and sedentary behaviors, leading to poor weight management (55). A population-based study has revealed significant relationships between adolescent aggressive and delinquent behavior problems with low self-esteem, parental and peer rejection, and low emotion warmth (56). It is important to note that new adjustments always happen as students grow older, such as the transition from primary to secondary school, which may cause new anxiety and emotional problems (57). These changes can affect weight status in children and adolescents. Our findings suggest the importance of considering psychological and emotional factors in weight status prediction and prevention.

Our feature selection analysis revealed the significant impact of socioeconomics factors, such as the location of students' residence and school, as well as the occupation of their mothers, on predicting long-term weight status. The geographical area where children live can greatly influenced their outdoor activities and the amount of time they spend on physical exercise. Moreover, environmental pollution has been linked to childhood weight problems. A systematic review has reported compelling evidence linking air pollution to a substantially increased risk of childhood obesity (58). Additionally, the role of mothers in Chinese family structure and parenting can impact the amount of time and attention devoted to a child's growth and development (59). These socio-economic factors have the potential to inspire new public health concerns and contribute to the prediction of long-term weight status.

### 4.2 Method selection and feature stability

Accurate and stable feature selection is crucial for obtaining a fixed set of common features while avoiding overfitting and ensuring a well predictive model. Previous studies have employed various feature selection techniques, but often without a comprehensive assessment of the stability and robustness of the selected features. One study conducted in Malaysia compared several methods to find the minimum number of features for predicting childhood obesity at 12 years old but did not validate the prediction results (21). Rehkopf et al. used a single method to get the relative importance of predictors for girls aged 9 or 10 years in BMI percentile changes (22). Gupta et al. also adopted a single feature selection approach to identify the top 20 important features without evaluating the stability of the feature set (24).

We opted for filter-univariate and embedded feature selection techniques, as they offer an effective and efficient feature selection process. The filter-univariate techniques, Chi-Square test and Information Gain, are more robust to outliers and can better handle mixed data types (categorical and numerical), compared to methods like ANOVA that rely on stricter data distribution assumptions. Furthermore, filter-univariate techniques are better able to capture non-linear relationships between features and the target variable, which is an important consideration in complex real-world datasets, compared to simpler techniques like Pearson correlation. While Wrapper feature selection methods fully consider feature dependencies, they are computationally expensive and time-consuming, as they require iteratively testing new feature subsets.

In the current study, we also conducted a comprehensive evaluation of the stability of our feature selection process, as this is often overlooked in previous studies. We assessed the robustness of the selected feature subsets using Jaccard's index, which measures the similarity of feature subsets across different iterations. Additionally, we examined the stability of the feature importance rankings and values using Spearman's rank correlation and Pearson's correlation, respectively. High values across these stability indices increase confidence in the identified predictors and ensure the generalizability of the developed predictive models (25).

In our study, XGBoost and RF models, utilizing a total of 38 features, outperformed the other ML approaches and LR in accurately predicting short- and long-term weight status. Moreover, XGBoost models using a stable and reduced set of features identified by the Chi-Square test demonstrated the better prediction performance in the multiclass models. Our large population-based study enabled us to make multiclass predictions for different years, by having an adequate number of children and adolescents in each weight status category. To the best of our knowledge, no other prediction models have been developed for temporal prediction of multiclass weight status in children and adolescents.

### 4.3 Limitations

This study has several limitations. First, the features collected from the SHS dataset did not capture all potentially relevant predictors of weight status in children and adolescents, such as family history of obesity, parental smoking, and lifestyle habits. Future research may incorporate these additional variables to enhance the predictive accuracy of weight status. Second, we had not tested our model's predictive performance in an external dataset. Evaluating the performance of the models on an independent dataset would be highly desirable to further assess the reliability and generalizability of the findings. Third, this study was conducted exclusively within the specific geographical context of Hong Kong, which has a relatively homogeneous Chinese population, accounting for over 91% of the sample. As such, the results may not fully represent the diverse populations and cultural contexts found in other regions. Additional research is needed to examine the transferability of the predictive models to more heterogeneous settings and populations with different cultural background.

## 5 Conclusions

We applied multiple feature selection frameworks to identify robust and minimal sets of features for predicting weight status in Hong Kong children and adolescents. The Chi-Square test method, combined with the XGBoost algorithm, provided the most robust and accurate results, with 6, 9, 13 features identified for 1-, 3-, and 5-year predictions, respectively. Weight, sex, height, total score of SEI, and age consistently emerged as key predictors across all short- and long-term predictions. Our study provides a comprehensive and robust list of predictors for weight status in children and adolescents in Hong Kong. This prediction model can be utilized by children, parents, and healthcare providers for early detection of weight problems, enabling timely intervention and prevention efforts.

## Data Availability

The datasets presented in this article are not readily available. The data supporting the conclusions of this study are available from the Student Health Services, Department of Health, Hong Kong SAR, but restrictions apply to the availability of these data, which were used under agreement for the current study, and so are not publicly available. Data are however available from the authors upon reasonable request and with permission of the Student Health Service, Department of Health, Hong Kong SAR. Requests to access the datasets should be directed to Thomas Wai Hung Chung, twhchung@dh.gov.hk.
