# Supplementary material for: Robust identification key predictors of short- and long-term weight status in children and adolescents by machine learning
Source: Front Public Health. 2024 Sep 24;12:1414046. doi: 10.3389/fpubh.2024.1414046 (PMC11458556; doi:10.3389/fpubh.2024.1414046)
Supplement: Supplementary file 1 [file Data_Sheet_1.docx]

Supplementary Material

| **Supplementary Table 1. Detailed list of collected data** |
| --- |

| **Assessment Item** | **Assessment Time** | **Assessment Content** | **Others** |
| --- | --- | --- | --- |
| Structured lifestyle questionnaire (for measuring physical health conditions) | For P4, P6, S2, S4, and S6 students | Assessing dietary and physical activity behavior by measuring frequency or duration.  It included the questions “breakfast eating habit (labeled as: old_A1)”, “sweetness preference during past 7 days (labeled as: old_A2)”, “junk food intake habit (labeled as: old_A3)”, “fruit/vegetable intake (labeled as: old_A4)”, and “milk consumption habit (labeled as: old_A5)”, “frequency of aerobic exercise each week (labeled as: old_A6_SEI, and old_A6_YSR)”, “hours of doing aerobic exercise each week (labeled as: old_A7)”, and “daily hours of TV viewing (labeled as: old_A8)”. | According to the responses of questions, they could be regarded as ordinal categorical variables.  The responses to the question on “Breakfast eating habit” were re-categorized to assess the frequency of breakfast eating at home with three response categories: (i) ‘home’, representing frequently eating at home, (ii) ‘rarely at home’, after combining the original categories of ‘fast food stall/cafeteria/restaurant’ and ‘some other places’, and (iii) ‘no breakfast at all’, representing never eating at home. |
| The 60-item self-reported Culture Free Self-Esteem Inventory for Children Questionnaire (SEI) | For P4 and P6 students | Evaluating psychological development.  SEI comprised one total score and four sub-domains: (i) self-esteem total score, (ii) parent-related self-esteem, denoting children’s perception on their family’s thoughts, (iii) parent-related self-esteem, denoting children’s perception on their family’s thoughts, (iv) school-related self-esteem, denoting children’s perception on their ability to achieve academic success. (1, 2) | Any scores of sub-domain item ≤ 2 were considered as “very-low”; (v) general self-esteem, denoting children’s overall perception of themselves, its score ≤ 7 was considered as “very-low”. (1) Low self-esteem was defined when children had a total score ≤ 19 or any domain with a “very-low” score. The SEI also contained a lie scale, and a scale score ≤ 2 indicated the corresponding child’s self-reported assessment was unreliable. (1)  This questionnaire had been assessed for reliability and validity in Hong Kong children and adolescents. |
| The Youth Self-Report (YSR) | For S4 and S6 students | Detecting various emotional and behavior problems.  It consisted of eight subscales: (i) withdrawal; (ii) somatic complaints; (iii) anxious/depressed; (iv) social problems; (v) thought problems; (vi) attention problems; (vii) delinquent behavior; (viii) aggressive behavior. (3, 4) | Scores above the local threshold in each syndrome indicated the respective problems, and a total score of over 78 in girls and 71 in boys indicated total psychological and behavior problems.(5)  This questionnaire had been assessed for reliability and validity in Hong Kong children and adolescents. |
| The 4-item Rutter Behavior Questionnaire (RBQ) | For P2, P4, and P6 students | Assessing potential behavioral problems.  The questionnaire was parent-reported and assessed behaviors from hyperactivity, conduct, and emotional disturbances perspectives. | A total score ≥ 19 on the RBQ indicated potential behavior problem in the child or adolescent.  This questionnaire had been assessed for reliability and validity in Hong Kong children and adolescents. |

| **Supplementary Table 2. Feature rank comparisons between data with and without k-NN imputed method for 1-year predictions** | | | | |  |
| --- | --- | --- | --- | --- | --- |
|  | Chi-Squre test | | | | |
| Feature rank | Dataset without missing value | | Dataset imputed by k-NN (k=3) | | |
| 1 | **wght** | 1154425.280 | **wght** | 1039795.1700 | |
| 2 | **age** | 142232.312 | **age** | 127889.4810 | |
| 3 | **hght** | 13934.033 | **hght** | 12562.0889 | |
| 4 | **sex** | 11390.315 | **sex** | 10241.8708 | |
| 5 | **SEI_total** | 2709.524 | **SEI_total** | 2451.4023 | |
| 6 | **YSR_thought** | 2242.077 | **YSR_thought** | 2000.2879 | |
| 7 | SEI_general | 2083.831 | SEI_general | 1885.0032 | |
| 8 | RBQ_emotion | 1674.440 | RBQ_emotion | 1516.1396 | |
| 9 | YSR_anxious | 1355.108 | YSR_anxious | 1233.4899 | |
| 10 | h_area_c | 1321.811 | h_area_c | 1191.3605 | |
| 11 | YSR_withdrawn | 1276.764 | YSR_withdrawn | 1161.2966 | |
| 12 | YSR_total | 1230.096 | YSR_total | 1095.7296 | |
| 13 | fat_ed_l | 1115.792 | fat_ed_l | 979.0421 | |
| 14 | SEI_academic | 1033.828 | SEI_academic | 923.9865 | |
| 15 | YSR_delinquent | 1001.885 | YSR_delinquent | 914.0015 | |
| 16 | dist_cde | 969.655 | dist_cde | 893.0145 | |
| 17 | fat_occ | 939.031 | fat_occ | 861.8692 | |
| 18 | YSR_somatic | 896.583 | YSR_somatic | 806.3389 | |
| 19 | old_A8 | 698.551 | RBQ_total | 641.8401 | |
| 20 | RBQ_total | 682.842 | old_A8 | 625.0100 | |
| 21 | SEI_parent | 669.018 | SEI_parent | 606.2832 | |
| 22 | SEI_social | 651.686 | SEI_social | 579.2729 | |
| 23 | old_A5 | 601.424 | old_A5 | 542.4950 | |
| 24 | RBQ_conduct | 581.376 | RBQ_conduct | 531.5972 | |
| 25 | mot_occ | 519.423 | mot_occ | 468.1606 | |
| 26 | YSR_aggressive | 499.817 | YSR_aggressive | 454.9404 | |
| 27 | RBQ_hyper | 486.293 | RBQ_hyper | 439.7730 | |
| 28 | YSR_social | 448.156 | YSR_social | 408.2493 | |
| 29 | house_cd | 431.325 | house_cd | 365.0687 | |
| 30 | mot_ed_l | 328.832 | mot_ed_l | 292.3774 | |
| 31 | YSR_attention | 315.202 | YSR_attention | 290.5299 | |
| 32 | old_A7 | 168.164 | old_A7 | 151.6857 | |
| 33 | old_A1 | 134.719 | old_A1 | 123.3975 | |
| 34 | old_A3 | 113.751 | old_A3 | 102.0268 | |
| 35 | old_A4 | 57.903 | old_A4 | 50.7066 | |
| 36 | old_A2 | 52.296 | old_A6_YSR | 46.0627 | |
| 37 | old_A6_YSR | 51.415 | old_A2 | 45.5532 | |
| 38 | old_A6_SEI | 36.439 | old_A6_SEI | 32.7937 | |
|  | Information Gain | | | | |
| Feature rank | Dataset without missing value | | Dataset imputed by k-NN (k=3) | | |
| 1 | **wght** | 0.118425 | **wght** | 0.481005 | |
| 2 | **hght** | 0.041274 | **hght** | 0.350603 | |
| 3 | **age** | 0.034346 | **age** | 0.297314 | |
| 4 | **YSR_total** | 0.029639 | **SEI_total** | 0.29114 | |
| 5 | **RBQ_total** | 0.029015 | **RBQ_total** | 0.289844 | |
| 6 | **SEI_total** | 0.026105 | **YSR_total** | 0.281217 | |
| 7 | SEI_general | 0.025407 | SEI_general | 0.272557 | |
| 8 | SEI_social | 0.025358 | SEI_academic | 0.263702 | |
| 9 | RBQ_emotion | 0.02417 | SEI_social | 0.262253 | |
| 10 | YSR_aggressive | 0.023124 | SEI_parent | 0.259507 | |
| 11 | SEI_academic | 0.018744 | YSR_aggressive | 0.259197 | |
| 12 | SEI_parent | 0.018362 | RBQ_hyper | 0.258931 | |
| 13 | YSR_anxious | 0.017501 | RBQ_emotion | 0.25683 | |
| 14 | RBQ_hyper | 0.017392 | YSR_anxious | 0.256115 | |
| 15 | old_A5 | 0.016341 | RBQ_conduct | 0.250998 | |
| 16 | RBQ_conduct | 0.015618 | old_A5 | 0.250864 | |
| 17 | old_A7 | 0.015527 | YSR_attention | 0.249425 | |
| 18 | YSR_attention | 0.015319 | old_A7 | 0.248043 | |
| 19 | YSR_withdrawn | 0.015176 | YSR_withdrawn | 0.247615 | |
| 20 | YSR_social | 0.013198 | YSR_somatic | 0.247102 | |
| 21 | YSR_somatic | 0.011987 | YSR_social | 0.24522 | |
| 22 | YSR_delinquent | 0.011814 | YSR_delinquent | 0.241762 | |
| 23 | old_A6_SEI | 0.01158 | old_A6_SEI | 0.238068 | |
| 24 | old_A8 | 0.010727 | YSR_thought | 0.237329 | |
| 25 | YSR_thought | 0.010679 | old_A8 | 0.234854 | |
| 26 | sex | 0.009661 | old_A6_YSR | 0.230647 | |
| 27 | fat_ed_l | 0.008498 | fat_occ | 0.226066 | |
| 28 | old_A6_YSR | 0.008325 | old_A4 | 0.22397 | |
| 29 | dist_cde | 0.006339 | old_A2 | 0.218901 | |
| 30 | mot_ed_l | 0.005395 | old_A3 | 0.214268 | |
| 31 | old_A4 | 0.00318 | fat_ed_l | 0.193977 | |
| 32 | old_A2 | 0.003009 | mot_ed_l | 0.187173 | |
| 33 | old_A3 | 0.002969 | old_A1 | 0.183019 | |
| 34 | mot_occ | 0.001376 | dist_cde | 0.17801 | |
| 35 | fat_occ | 0.000492 | h_area_c | 0.176605 | |
| 36 | house_cd | 0 | mot_occ | 0.151116 | |
| 37 | h_area_c | 0 | house_cd | 0.15023 | |
| 38 | old_A1 | 0 | sex | 0.129817 | |
|  | XGBoost | | | | |
| Feature rank | Dataset without missing value | | Dataset imputed by k-NN (k=3) | | |
| 1 | **age** | 0.1196989 | **age** | 0.22039667 | |
| 2 | **wght** | 0.11466854 | **wght** | 0.19098972 | |
| 3 | **hght** | 0.09763026 | **sex** | 0.14989223 | |
| 4 | **old_A1** | 0.03305924 | **hght** | 0.07552929 | |
| 5 | **sex** | 0.02907792 | **old_A1** | 0.06145086 | |
| 6 | **old_A7** | 0.0280546 | **old_A3** | 0.0115505 | |
| 7 | old_A5 | 0.02761628 | old_A7 | 0.01036175 | |
| 8 | old_A2 | 0.02592565 | old_A2 | 0.01023634 | |
| 9 | YSR_aggressive | 0.02548261 | old_A4 | 0.01020571 | |
| 10 | YSR_attention | 0.02194667 | house_cd | 0.0101558 | |
| 11 | h_area_c | 0.02174309 | mot_ed_l | 0.00986076 | |
| 12 | RBQ_conduct | 0.02119369 | h_area_c | 0.00986008 | |
| 13 | old_A4 | 0.02041997 | old_A6_YSR | 0.00981821 | |
| 14 | RBQ_total | 0.01971207 | fat_ed_l | 0.00972566 | |
| 15 | YSR_social | 0.01953755 | old_A6_SEI | 0.00944383 | |
| 16 | YSR_thought | 0.01940449 | YSR_total | 0.00935059 | |
| 17 | old_A8 | 0.01931852 | mot_occ | 0.00929955 | |
| 18 | mot_occ | 0.0192974 | dist_cde | 0.00928862 | |
| 19 | old_A6_YSR | 0.01891438 | old_A8 | 0.00915211 | |
| 20 | mot_ed_l | 0.01832606 | SEI_social | 0.00912395 | |
| 21 | dist_cde | 0.01817529 | SEI_academic | 0.00900317 | |
| 22 | SEI_social | 0.01769969 | old_A5 | 0.00887204 | |
| 23 | **YSR_withdrawn** | 0.01734173 | YSR_social | 0.00883196 | |
| 24 | SEI_total | 0.01733463 | RBQ_conduct | 0.00877862 | |
| 25 | fat_occ | 0.01649254 | YSR_withdrawn | 0.00872515 | |
| 26 | YSR_delinquent | 0.01637194 | YSR_delinquent | 0.00871916 | |
| 27 | RBQ_emotion | 0.01603129 | YSR_thought | 0.00866899 | |
| 28 | old_A3 | 0.01554756 | SEI_parent | 0.00866609 | |
| 29 | YSR_anxious | 0.01544995 | fat_occ | 0.00860529 | |
| 30 | YSR_somatic | 0.01542235 | RBQ_emotion | 0.00859587 | |
| 31 | RBQ_hyper | 0.01480694 | SEI_general | 0.00852827 | |
| 32 | old_A6_SEI | 0.01453922 | SEI_total | 0.00844378 | |
| 33 | SEI_general | 0.01450091 | RBQ_hyper | 0.00842138 | |
| 34 | **SEI_academic** | 0.0145 | YSR_attention | 0.00842026 | |
| 35 | YSR_total | 0.01430055 | YSR_aggressive | 0.00834522 | |
| 36 | SEI_parent | 0.01378711 | YSR_somatic | 0.00829775 | |
| 37 | house_cd | 0.01334669 | YSR_anxious | 0.00826768 | |
| 38 | fat_ed_l | 0.01332537 | RBQ_total | 0.00811714 | |
|  | Random Forest | | | | |
| Feature rank | Dataset without missing value | | Dataset imputed by k-NN (k=3) | | |
| 1 | **wght** | 0.24311665 | **wght** | 0.2978678 | |
| 2 | **hght** | 0.08062912 | **age** | 0.20442223 | |
| 3 | **age** | 0.07798084 | **hght** | 0.10926075 | |
| 4 | **sex** | 0.02919642 | **sex** | 0.0312556 | |
| 5 | **old_A7** | 0.02607056 | **old_A1** | 0.02173105 | |
| 6 | **old_A6_SEI** | 0.02360017 | **old_A3** | 0.01535357 | |
| 7 | RBQ_total | 0.02353943 | house_cd | 0.01231822 | |
| 8 | RBQ_emotion | 0.02255416 | old_A7 | 0.01211842 | |
| 9 | YSR_delinquent | 0.02222329 | old_A6_SEI | 0.01134211 | |
| 10 | YSR_withdrawn | 0.02178199 | old_A2 | 0.01115093 | |
| 11 | YSR_aggressive | 0.02161637 | RBQ_total | 0.01084482 | |
| 12 | old_A8 | 0.02138957 | YSR_total | 0.01084 | |
| 13 | RBQ_hyper | 0.02085845 | SEI_total | 0.01073054 | |
| 14 | SEI_general | 0.02040665 | old_A6_YSR | 0.01044148 | |
| 15 | old_A6_YSR | 0.0202347 | old_A8 | 0.01038232 | |
| 16 | old_A3 | 0.02014163 | h_area_c | 0.01035519 | |
| 17 | YSR_anxious | 0.02006436 | dist_cde | 0.0102631 | |
| 18 | dist_cde | 0.01993529 | SEI_general | 0.01009503 | |
| 19 | SEI_parent | 0.01985492 | YSR_aggressive | 0.0100724 | |
| 20 | YSR_total | 0.01823673 | fat_occ | 0.01005138 | |
| 21 | h_area_c | 0.01801126 | old_A4 | 0.01000466 | |
| 22 | YSR_thought | 0.01765638 | old_A5 | 0.00992624 | |
| 23 | YSR_social | 0.01671967 | SEI_social | 0.00987003 | |
| 24 | YSR_somatic | 0.01601214 | YSR_anxious | 0.00977192 | |
| 25 | YSR_attention | 0.01554094 | RBQ_hyper | 0.00972732 | |
| 26 | old_A5 | 0.01547505 | SEI_academic | 0.00970427 | |
| 27 | SEI_academic | 0.01510115 | YSR_delinquent | 0.00964608 | |
| 28 | RBQ_conduct | 0.01329805 | YSR_thought | 0.00949119 | |
| 29 | fat_occ | 0.01302275 | SEI_parent | 0.00949061 | |
| 30 | fat_ed_l | 0.01257061 | RBQ_conduct | 0.009421 | |
| 31 | SEI_social | 0.01193531 | YSR_somatic | 0.00929713 | |
| 32 | old_A4 | 0.01155171 | YSR_social | 0.00929208 | |
| 33 | SEI_total | 0.01080106 | YSR_attention | 0.00927122 | |
| 34 | mot_ed_l | 0.01002759 | RBQ_emotion | 0.00925754 | |
| 35 | old_A1 | 0.00975365 | fat_ed_l | 0.00922559 | |
| 36 | mot_occ | 0.00843819 | mot_ed_l | 0.00915526 | |
| 37 | house_cd | 0.00732316 | YSR_withdrawn | 0.00913821 | |
| 38 | old_A2 | 0.00333002 | mot_occ | 0.00741271 | |

| **Supplementary Table 3.** Hyperparameter tuning for machine learning models | | | | |
| --- | --- | --- | --- | --- |
| Machine Learning Model | Hyperparameter | Range/Values Tested | Best Value for the Training Phase | Best Value for the Temporal Test Phase |
| eXtreme Gradient Boosting | n_estimators | 10, 20, 50, 100, 200, 500 | 100 | 100 |
|  | max_depth | 10, 20, 30, 40, 50 | 20 | 20 |
|  | num_boost_round | 10, 20, 30, 40, 60, 80 | 60 | 60 |
|  | learning_rate | 0.01, 0.1, 0.3 | 0.1 | 0.1 |
|  | min_child_weight | 1, 3, 5 | 3 | 3 |
| Random Forest | n_estimators | 10, 20, 50, 100, 200, 500 | 100 | 20 |
|  | max_depth | 10, 20, 30, 40, 50 | 40 | 40 |
|  | max_features | 3, 6, 9, 12, 15, 18 | 12 | 12 |
|  | min_samples_split | 2, 5, 10 | 5 | 5 |
| Decision Tree | max_depth | 10, 20, 30, 40, 50 | 10 | 10 |
|  | min_samples_split | 2, 5, 10 | 5 | 2 |
| Support Vector Machine | C | 0.1, 1, 10 | 1 | 1 |
|  | gamma | 0.01, 0.1, 1 | 0.1 | 0.1 |
|  | kernel | linear', 'rbf', 'poly', 'sigmoid' | linear' | linear' |
| k-Nearest Neighbors | n_neighbors | 3, 5, 7 | 3 | 3 |
|  | metric | 'euclidean', 'manhattan', 'minkowski' | 'euclidean' | 'euclidean' |
| Logistic Regression | C | 0.1, 1, 10 | 1 | 1 |
|  | penalty | 'l1', 'l2' | 'l2' | 'l2' |
|  | solver | 'liblinear', 'lbfgs', 'saga' | 'liblinear' | 'liblinear' |

| **Supplementary Table 4.** Baseline socioeconomic characteristics of training and temporal test sets | | | |
| --- | --- | --- | --- |
| **Characteristics** | | **Training set**  N_1_=3,727,390 | **Temporal Test Set (Academic cohorts of 2010/11-2014/15)** N2=135,430 |
|  |  |  |  |
| **Educational Level of Student's Father** | | | |
|  | Missing value | 85,730 (2.3) | 2,162 (1.6) |
|  | No Schooling | 22,364 (0.6) | 797 (0.6) |
|  | Kindergarten | 7,455 (0.2) | 0 |
|  | Primary | 615,019 (16.5) | 23,217 (17.1) |
|  | Lower Secondary | 786,479 (21.1) | 33,118 (24.5) |
|  | Upper Secondary | 1,531,957 (41.1) | 51,896 (38.3) |
|  | Matriculation | 186,370 (5.0) | 4,666 (3.4) |
|  | Tertiary (Non-degree Course) | 100,640 (2.7) | 6,942 (5.1) |
|  | Tertiary (Degree Course) | 391,376 (10.5) | 12,633 (9.3) |
| **Educational Level of Student's Mother** | | | |
|  | Missing value | 67,093 (1.8) | 0 |
|  | No Schooling | 26,092 (0.7) | 1,934 (1.4) |
|  | Kindergarten | 7,455 (0.2) | 0 |
|  | Primary | 544,199 (14.6) | 25,379 (18.7) |
|  | Lower Secondary | 812,571 (21.8) | 30,955 (22.9) |
|  | Upper Secondary | 1,733,236 (46.5) | 65,325 (48.2) |
|  | Matriculation | 137,913 (3.7) | 1,593 (1.2) |
|  | Tertiary (Non-degree Course) | 111,822 (3.0) | 4,438 (3.3) |
|  | Tertiary (Degree Course) | 287,009 (7.7) | 5,804 (4.3) |
| **Occupation of Student's Father** | | | |
|  | Missing value | 141,641 (3.8) | 2,167 (3.9) |
|  | Managers and Administrators | 391,376 (10.5) | 17,606 (11.0) |
|  | Professionals | 175,187 (4.7) | 6,501 (4.8) |
|  | Associate Professionals | 227,371 (6.1) | 8,397 (6.2) |
|  | Clerks | 383,921 (10.3) | 13,137 (9.6) |
|  | Service Workers and Shop Sales Workers | 697,022 (18.7) | 25,325 (18.6) |
|  | Craft and Related Workers | 574,018 (15.4) | 21,533 (15.9) |
|  | Plant & Machine Operators and Assemblers | 577,745 (15.5) | 20,992 (15.4) |
|  | Elementary Occupations | 451,014 (12.1) | 15,304 (11.6) |
|  | Unemployed | 108,094 (2.9) | 4,469 (3.3) |
| **Occupation of Student's Mother** | | | |
|  | Missing value | 55,911 (1.5) | 1,083 (1.3) |
|  | Managers and Administrators | 111,822 (3.0) | 3,927 (2.8) |
|  | Professionals | 67,093 (1.8) | 2,573 (1.6) |
|  | Associate Professionals | 164,005 (4.4) | 5,823 (4.3) |
|  | Clerks | 622,474 (16.7) | 22,888 (16.7) |
|  | Service Workers and Shop Sales Workers | 469,651 (12.6) | 15,574 (11.2) |
|  | Craft and Related Workers | 41,001 (1.1) | 2,844 (1.1) |
|  | Plant & Machine Operators and Assemblers | 18,637 (0.5) | 542 (0.4) |
|  | Elementary Occupations | 156,551 (4.2) | 13,001 (4.0) |
|  | Unemployed | 2,031,428 (54.5) | 67,173 (55.7) |
| **Type of Housing** | | | |
|  | Missing value | 55,911 (1.5) | 2,167 (1.6) |
|  | Block-Self-contained | 1,520,775 (40.8) | 55,662 (41.2) |
|  | Block-non-Self-contained | 82,002 (2.2) | 2,844 (2.0) |
|  | Housing Authority Home Ownership Estate | 618,747 (16.6) | 22,888 (16.9) |
|  | Housing Authority/Society Blocks | 1,248,676 (33.5) | 45,504 (33.8) |
|  | Village Houses | 156,550 (4.2) | 5,146 (3.6) |
|  | Institution | 44,729 (1.2) | 1,354 (1.0) |
| **Student's Home District** | |  |  |
|  | Missing value | 4,236 (0.06) | 0 |
|  | Central & Western | 203,143 (5.45) | 6,636 (4.90) |
|  | Wan Chai | 43,238 (1.16) | 1,612 (1.19) |
|  | Eastern | 457,723 (12.28) | 16,929 (12.50) |
|  | Southern | 159,160 (4.27) | 5,837 (4.31) |
|  | Yau Tsim Mong | 119,276 (3.20) | 4,104 (3.03) |
|  | Sham Shui Po | 147,977 (3.97) | 5,431(4.01) |
|  | Kowloon City | 161,023 (4.32) | 5,905 (4.36) |
|  | Wong Tai Sin | 228,116 (6.12) | 8,234 (6.08) |
|  | Kwun Tong | 321,301 (8.62) | 12,080 (8.92) |
|  | Tsuen Wan | 110,703 (2.97) | 3,318 (2.45) |
|  | Tuen Mun | 231,471 (6.21) | 8,464 (6.25) |
|  | Yuen Long | 382,803 (10.27) | 14,220 (10.50) |
|  | North | 218,798 (5.87) | 7,598 (5.61) |
|  | Tai Po | 163,260 (4.38) | 6,000 (4.43) |
|  | Sai Kung | 222,898 (5.98) | 8,045 (5.94) |
|  | Sha Tin | 331,738 (8.90) | 12,189 (9.00) |
|  | Kwai Tsing | 177,424 (4.76) | 7,137 (5.27) |
|  | Islands | 43,610 (1.17) | 1,585 (1.17) |
|  | China | 1,118 (0.03) | 68 (0.03) |
|  | Macau | 373 (0.00) | 41 (0.00) |
| **Student's School District** | |  |  |
|  | Missing value | 745 (0.02) | 0 |
|  | Central & Western | 190,470 (5.11) | 7,259 (5.36) |
|  | Wan Chai | 188,233 (5.05) | 6,595 (4.87) |
|  | Eastern | 378,330 (10.15) | 14,139 (10.44) |
|  | Southern | 130,831 (3.51) | 5,336 (3.94) |
|  | Yau Tsim Mong | 155,432 (4.17) | 5,661 (4.18) |
|  | Sham Shui Po | 174,442 (4.68) | 6,040 (4.46) |
|  | Kowloon City | 295,209 (7.92) | 10,834 (8.00) |
|  | Wong Tai Sin | 229,234 (6.15) | 7,679 (5.67) |
|  | Kwun Tong | 251,972 (6.76) | 9,101 (6.72) |
|  | Tsuen Wan | 225,134 (2.52) | 2,871 (2.12) |
|  | Tuen Mun | 225,134 (6.04) | 8,410 (6.21) |
|  | Yuen Long | 383,921 (10.30) | 13,665 (10.09) |
|  | North | 201,652 (5.41) | 7,015 (5.18) |
|  | Tai Po | 162,141 (4.35) | 6,108 (4.51) |
|  | Sai Kung | 172,578 (4.63) | 5,986 (4.42) |
|  | Sha Tin | 306,764 (8.23) | 11,850 (8.75) |
|  | Kwai Tsing | 143,877 (3.86) | 5,661 (4.18) |
|  | Islands | 42,492 (1.14) | 1,246 (0.92) |

Data are n (%) unless otherwise stated.

**References**

1. Battle J. Culture-Free Self-Esteem Inventories 2nd ed. Austin, TX: Pro-Ed. 1992.

2. Chan YY. The normative data and factor structure of the culture-free self-esteem inventory-form a-second edition in Hong Kong adolescents. Hong Kong, CN: The University of Hong Kong; 2002. HKU Theses Online (HKUTO): b2974025.

3. Ivanova MY, Achenbach TM, Rescorla LA, et al. The general-izability of the youth self-report syndrome structure in 23 societies. J Consult Clin Psychol. 2007;75(5):729–738.

4. Leung PW, Kwong SL, Tang CP, et al. Test–retest reliability and criterion validity of the Chinese version of CBCL, TRF, and YSR. J Child Psychol Psychiatry. 2006;47(9):970–973.

5. Leung P, Lee C, Ho T, Hung S, Tang C. Achenbach Hong Kong norm: YSR proﬁle for Hong Kong boys & girls. Unpublished manuscript The Chinese University of Hong Kong. Hong Kong, China 1998.
